# Identification of oxidative stress-related biomarkers associated with the development of acute-on-chronic liver failure using bioinformatics

**DOI:** 10.1038/s41598-023-44343-9

**Published:** 2023-10-10

**Authors:** Zongyi Zhu, Huiqing Jiang

**Affiliations:** 1 Department of Gastroenterology, Hebei Key Laboratory of Gastroenterology, Hebei Institute of Gastroenterology, Hebei Clinical Research Center for Digestive Diseases, The Second Hospital of Hebei Medical University, Shijiazhuang, Hebei China; 2 Department of Gastroenterology, Weixian People’s Hospital, Xingtai, Hebei China

**Keywords:** Diagnostic markers, Prognostic markers, Computational biology and bioinformatics

## Abstract

Acute-on-chronic liver failure (ACLF) is a serious stage of chronic liver disease with high short-term mortality and no definitely effective treatment. Oxidative stress (OS) is involved in the development of ACLF. OS-related genes targeted therapy may provide additional assistance for the treatment of ACLF. ACLF related gene sets and oxidative stress-related genes (OSGs) were respectively downloaded from gene expression omnibus (GEO) database and GeneCards database for integrated bioinformatics analyses (functional enrichment, weighted gene co-expression network and immune cells infiltration). Immune-related differentially expressed oxidative stress-related genes (DEOSGs) in ACLF were used for construction of protein–protein interaction (PPI) network in which hub genes were screened out. Hub genes with consistently good diagnostic or prognostic value for ACLF in four gene sets were named as key genes. DEOSGs were significantly enriched in biological process and signaling pathways related to inflammation, immune response and oxidative stress. Six key genes (MPO, CCL5, ITGAM, TLR2, TLR4, and TIMP1) were identified and found to be highly correlated with immune response and metabolic process. This study deepened our understanding of the impact of oxidative stress on the pathogenesis and prognosis of ACLF and provided more insights into the prediction of prognosis and molecular targeted therapy in ACLF.

## Introduction

Acute-on-chronic liver failure (ACLF) is a distinct syndrome characterized by acute decompensation of chronic liver disease, multiple organ failures and high short-term mortality^1^. The pathophysiological mechanisms underlying ACLF mainly include intense systemic inflammation, immune dysfunction, mitochondrial dysfunction, metabolic changes and oxidative stress^2–4^. Up to now, there are no definitely effective treatment strategies for ACLF, except liver transplantation. The main principle in the management of ACLF is to treat the precipitating event or associated complications and provide organ support therapy^5,6^. There is an urgent need to further explore the pathogenesis underlying the development and progression of ACLF in order to develop more effective treatment strategies and reduce the high mortality in this entity.

Oxidative stress-related biomarkers may have prognostic and therapeutic values for ACLF. Current advancements in the understanding of the pathophysiological basis underlying ACLF suggest that a hyper-reactive systemic inflammatory response triggered by pathogen-associated molecular patterns (PAMPs) or damaged-associated molecular patterns (DAMPs) is the critical driver of tissue damage and organ injury in patients with acutely decompensated chronic liver disease leading to the development of ACLF^7^. Systemic inflammation can result in an early activation and later paralysis of immune system^2,3^. In addition, systemic inflammation inhibits oxidative phosphorylation (OXPHOS) in mitochondria and the translocation of fatty acids into mitochondria, which is required for fatty acid β-oxidation^4,8,9^, subsequently not only resulting in impairment of ATP production but also leakage of electrons at the electron transport chain (ETC) and enhanced production of reactive oxygen species (ROS) causing oxidative damage to DNA, proteins and lipid membranes^3^. If the antioxidant capacity is insufficient and/or the incurred damage is not efficiently repaired, ROS may ultimately lead to oxidative stress (OS)-related damage, such as cell apoptosis, tissue damage and organ dysfunction which is the pathophysiological basis for the development and progression of ACLF^3^. As OS is highly associated with the initiation and progression of ACLF, identification and comprehensive analysis of the oxidative stress-related genes and their regulatory networks may not only deepen our understanding about the impact of gene regulation on the severity of oxidative stress, but also provide promising candidate biomarkers used as therapeutic targets and prognostic predictors for ACLF. In fact, some immune or metabolism-related genes have already been proven to be highly related to the pathogenesis, severity and prognosis of ACLF ^4,10,11^. However, there is no study to explore the role of oxidative stress-related genes in the development and progression of ACLF.

In recent years, with the advancement of microarray and high throughput technology, the bioinformatics methods were used more and more to reveal the pathophysiological mechanisms underlying various diseases at the genomic level^12^. Numerous biomarkers which can be used in disease diagnosis, targeted therapy and prognosis prediction were identified using bioinformatics methods. In this study, we aimed to use integrated bioinformatics method to identify the oxidative stress-related genes and their regulatory networks, which might provide new perspectives for the molecular targeted therapy and prediction of prognosis in ACLF.

## Materials and methods

### Data collection

**Figure 1:**
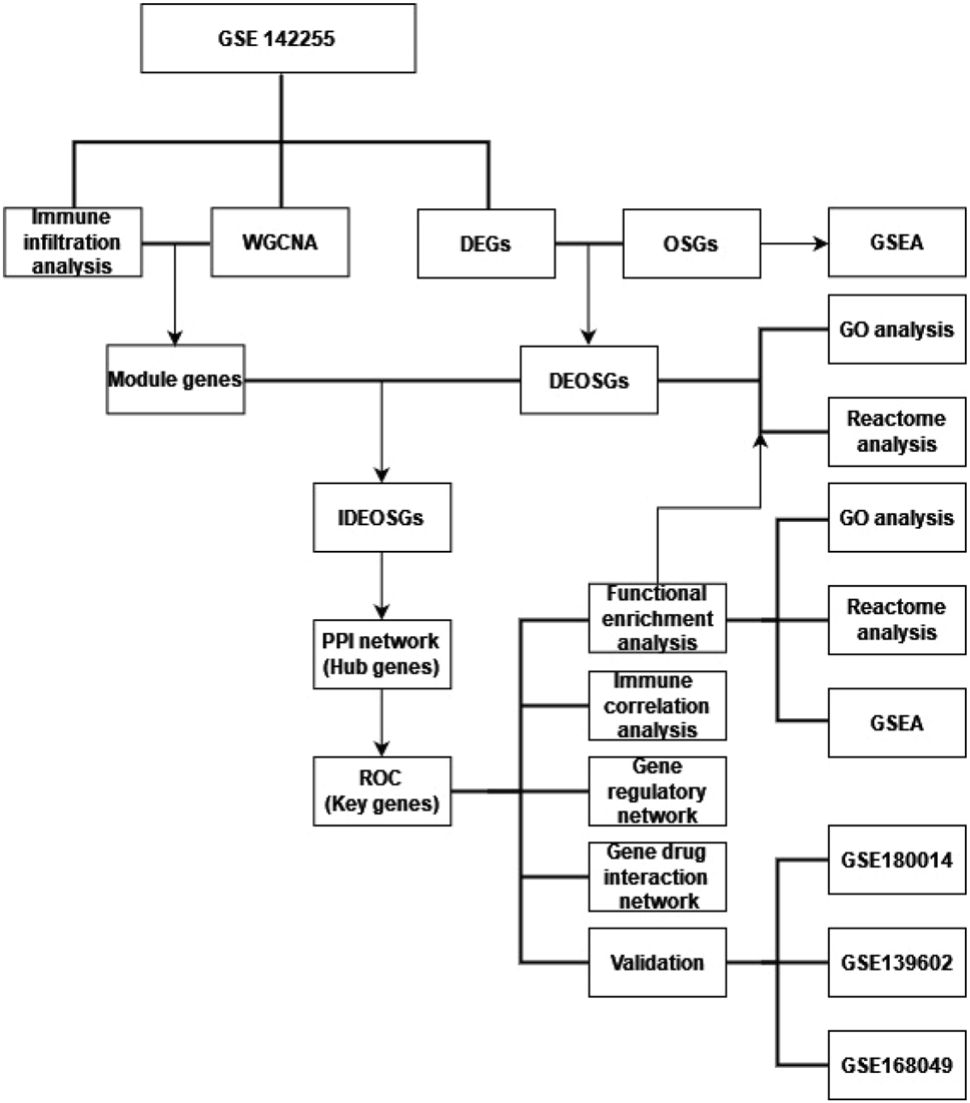
Flowchart of this study. *DEGs* differentially expressed genes, *DEOSGs* differentially expressed oxidative stress related genes, *GO* gene ontology, *GSEA* gene set enrichment analysis, *IDEOSGs* immune related DEOSGs, *OSGs* oxidative stress related genes, *PPI* protein–protein interaction network, *ROC* receiver operating characteristic curve, *WGCNA* weighted gene co-expression network analysis.

Four transcriptome datasets (GSE142255 (https://www.ncbi.nlm.nih.gov/geo/query/acc.cgi?acc=GSE142255), GSE180014, GSE139602 and GSE168049) related to ACLF were downloaded from Gene Expression Omnibus (GEO) database (https://www.ncbi.nlm.nih.gov/geo/)^13^. GSE142255 contained array gene profiling of immune cells in whole-blood from 17 ACLF patients and 7 healthy controls (HCs). GSE180014 contained RNA-seq transcriptomics profiling in peripheral leukocytes from 8 ACLF patients and 5 HCs. GSE139602 contained array profiling on liver biopsies from 8 ACLF patients and 6 HCs. GSE168049 contained array profiling in peripheral blood mononuclear cells (PBMCs) from 8 dead hepatitis B virus-related ACLF (HBV-ACLF) patients and 8 live HBV-ACLF patients. GSE142255 was used as the derivation set to screen the hub genes with most connectivity in the protein–protein interaction (PPI) network. GSE180014 and GSE139602 were used to validate the role of hub genes in the diagnosis of ACLF from the perspectives of peripheral leukocytes and liver biopsy, respectively, while GSE168049 was used to validate the role of hub genes in predicting the prognosis of patients with ACLF. 1093 oxidative stress-related genes (OSGs) were obtained from the GeneCards database (https://www.genecards.org/) with relevance score ≥ 7. The workflow of this study was shown in Fig. 1.

### Identification of differentially expressed genes

The “limma” package in R language software was applied to screen the differentially expressed genes (DEGs) between patients with ACLF and healthy controls (HC) with the criterion of |log 2 Fold Change| > 0.58 and adjusted *P* value < 0.05. Differentially expressed oxidative stress-related genes (DEOSGs) between patients with ACLF and HC were obtained by the intersection of DEGs and OSGs with the “VennDiagram” package in R.

#### Functional enrichment analysis

Gene Ontology (GO) (http://geneontology.org/) enrichment analysis and Reactome (https://reactome.org/) enrichment analysis were performed to reveal the biological functions and pathways related to DEOSGs and key genes using the “clusterProfiler” R package. An adjusted *P* value < 0.05 was considered statistically significant.

#### Gene set enrichment analysis

Gene set enrichment analysis (GSEA) was performed using the “clusterProfiler”, “enrichplot”, “pathwork” and “DOSE” packages in R and an online analysis platform: Sanger box (http://vip.sangerbox.com). According the aim of analysis, the reference gene sets included c5.go.bp.v2023.1.Hs.symbols.gmt, c2.cp.reactome.v2023.1.Hs.symbols.gmt, c2.cp.kegg.v7.4.symbols.gmt and blood transcriptional modules (BTMs). BTMs are molecular signatures constructed using transcriptomic data in peripheral-blood mononuclear cells from individuals under various immunological stimuli developed by Li et al.^14^. The input gene expression matrix was Robust Multichip Average (RMA) normalized micro array profiling in GSE142255 data set. The parameter settings were as follows: minimum gene set 5, maximum gene set 5000 and 1000 resamplings. Normalized enrichment score (NES) was used to indicate the strength of enrichment. |NES| ≥ 1.0 and adjusted or nominal P-value < 0.05 were defined as statistical significance.

### Immune infiltration analysis

Proportions of 22 types of immune cells in samples from GSE142255 were obtained using the CIBERSORT algorithm. The “tinyarray” R package was used to compare the levels of 22 types of immune cells between ACLF patients and healthy subjects. Differentiated infiltrating immune cells were identified by *P* value < 0.05 in the Kruskal test.

#### Weighted gene co-expression network analysis

Weighted gene co-expression network analysis (WGCNA), a system biology strategy adopted for identifying co-expressed gene modules and exploring their correlation with disease phenotypes, was used to identify the modules genes highly correlated with the significantly differentially infiltrating immune cells between ACLF and HC. To omit the unqualified genes and samples, we used genes with a median absolute deviation (MAD) of the top 5000 for subsequent analysis. The best soft-thresholding power β was chosen according to the criteria of approximate scale-free topology. Genes with identical expression profiles were classified into gene modules using average linkage hierarchical clustering with a topological overlap matrix (TOM) based dissimilarity metric. The correlation coefficient between modules and differentiated infiltrating immune cells were calculated and modules with a high correlation coefficient (r > 0.7, *P* < 0.001) were identified as immune-related modules.

#### Identification of immune-related oxidative stress DEGs

The intersection between DEOSGs and genes in immune-related modules was defined as immune-related DEOSGs for subsequent analysis.

### Construction of protein–protein interaction network and screening of hub genes

The immune-related DEOSGs were uploaded to STRING database (https://cn.string-db.org/) to construct the protein–protein interaction (PPI) network with an interaction score of 0.4. The PPI network was then imported to Cytoscape (http://cytoscape.org/.ver.3.9.1) software, an open-source network visualization platform for any molecular compositions and interaction systems^15^. The most densely connected cluster in PPI was identified using the molecular complex detection technology (MCODE) plug-in in Cytoscape: K-core = 2, degree cutoff = 2, max depth = 100, and node score cutoff = 0.2. Hub genes in the most densely connected cluster were screened out by the Maximal Clique Centrality (MCC) algorithm of CytoHubba plug-in, which can recognize the interaction degree of candidate genes in a network through 11 topological algorithms^16^.

### Identification of key genes

Diagnostic and prognostic value of hub genes for ACLF were determined by performing receiver operating characteristic curve (ROC) and calculating the area under the curve (AUC). Hub genes with a AUC > 0.7 indicating good diagnostic and prognostic value were named as key genes.

### Exploration of transcription factors and miRNAs regulating key genes

Transcription factors (TFs) and micro RNAs (miRNAs) regulating key genes were obtained from JASPAR database and TarBase (v8.0) database, respectively, using NetworkAnalyst, an online analysis platform which integrated publicly available and unrestricted access to multiple databases for the network analysis of genes and multiple molecular components^17^. Furthermore, the TF-gene and miRNA-gene interaction network were visualized with Cytoscape.

### Identification of potentially effective drugs targeting key genes

Potentially effective drugs targeting key genes were obtained from Drug-gene interactions database (DGIdb)^18^ (https://dgidb.genome.wustl.edu/) and visualized with Cytoscape.

### Statistical analysis

All statistical analyses were conducted using the R programming language. Comparison of data between different groups were performed with independent-samples t-test or Kruskal test. A *P*-value less than 0.05 was considered statistically significant.

## Results

### Identification of DEGs and DEOSGs

**Figure 2:**
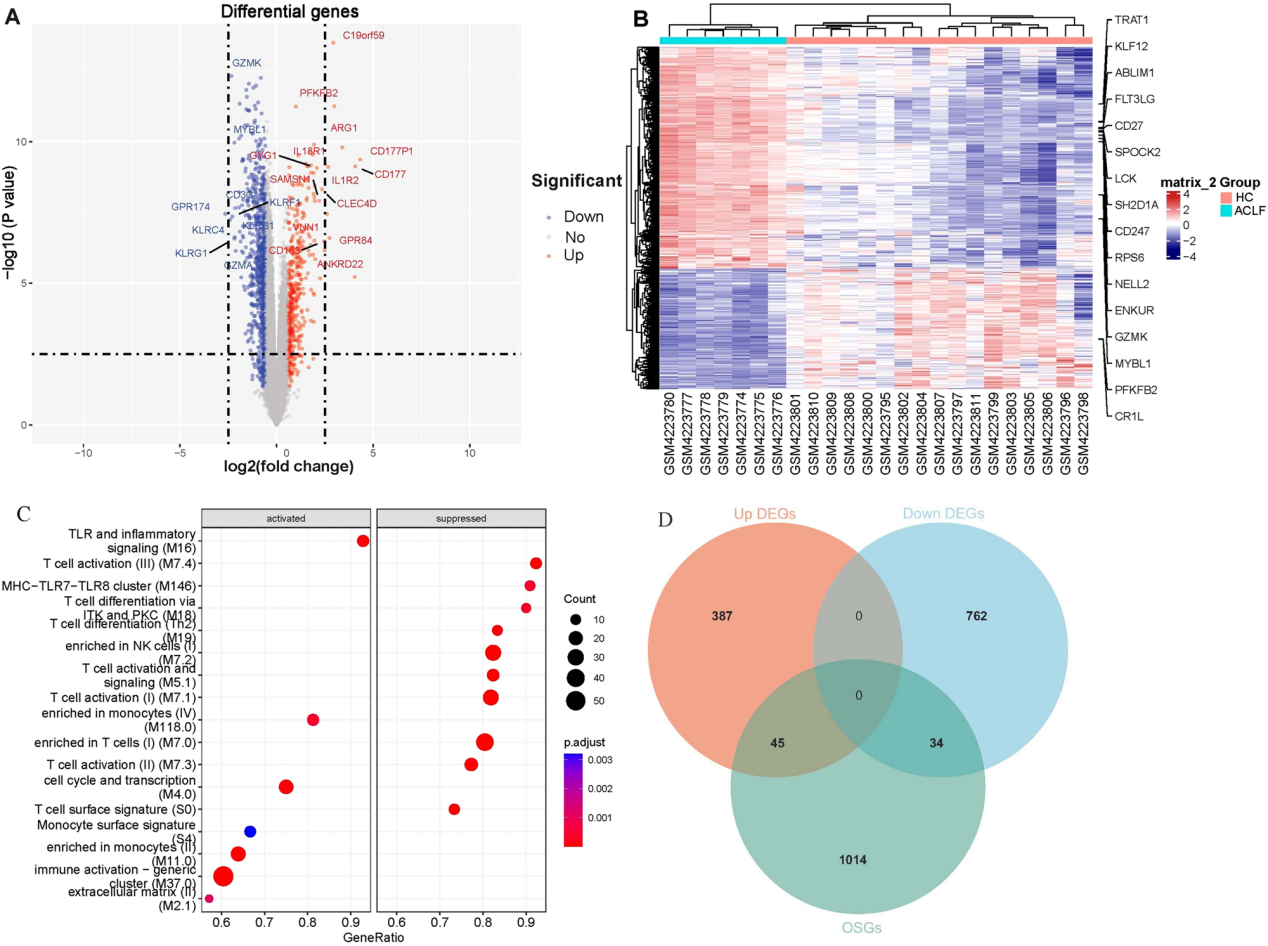
DEGs and DEOSGs in ACLF. (**A**) Volcano plot displaying DEGs. (**B**) Heatmap displaying DEGs. (**C**) Immune cells and signaling pathways enrichment analysis for DEGs. (**D**) Venn diagram displaying DEOSGs.

A total of 1228 differentially expressed genes (DEGs) between ACLF patients and healthy subjects were screened out from data set GSE142255, including 432 up-regulated genes and 796 down-regulated genes (Fig. 2A,B). To further identify the source and pathways of these DEGs, we performed GSEA using BTMs as reference gene sets. The result showed that up-regulated DEGs were significantly enriched in cell lines or pathways related to TLR and inflammatory signaling, monocytes, cell cycle and transcription, monocyte surface signature, immune activation-generic cluster and extracellular matrix, while down-regulated DEGs were significantly enriched in cell lines or pathways related to T cell activation, signaling and differentiation, T cell surface signature, NK cells and MHC-TLR7-TLR8 cluster (Fig. 2C). After the intersection between 1228 DEGs and 1093 oxidative stress-related genes (OSGs), 79 differentially expressed oxidative stress-related genes (DEOSGs) were obtained (Fig. 2D).

### Functional enrichment analysis for DEOSGS

**Figure 3:**
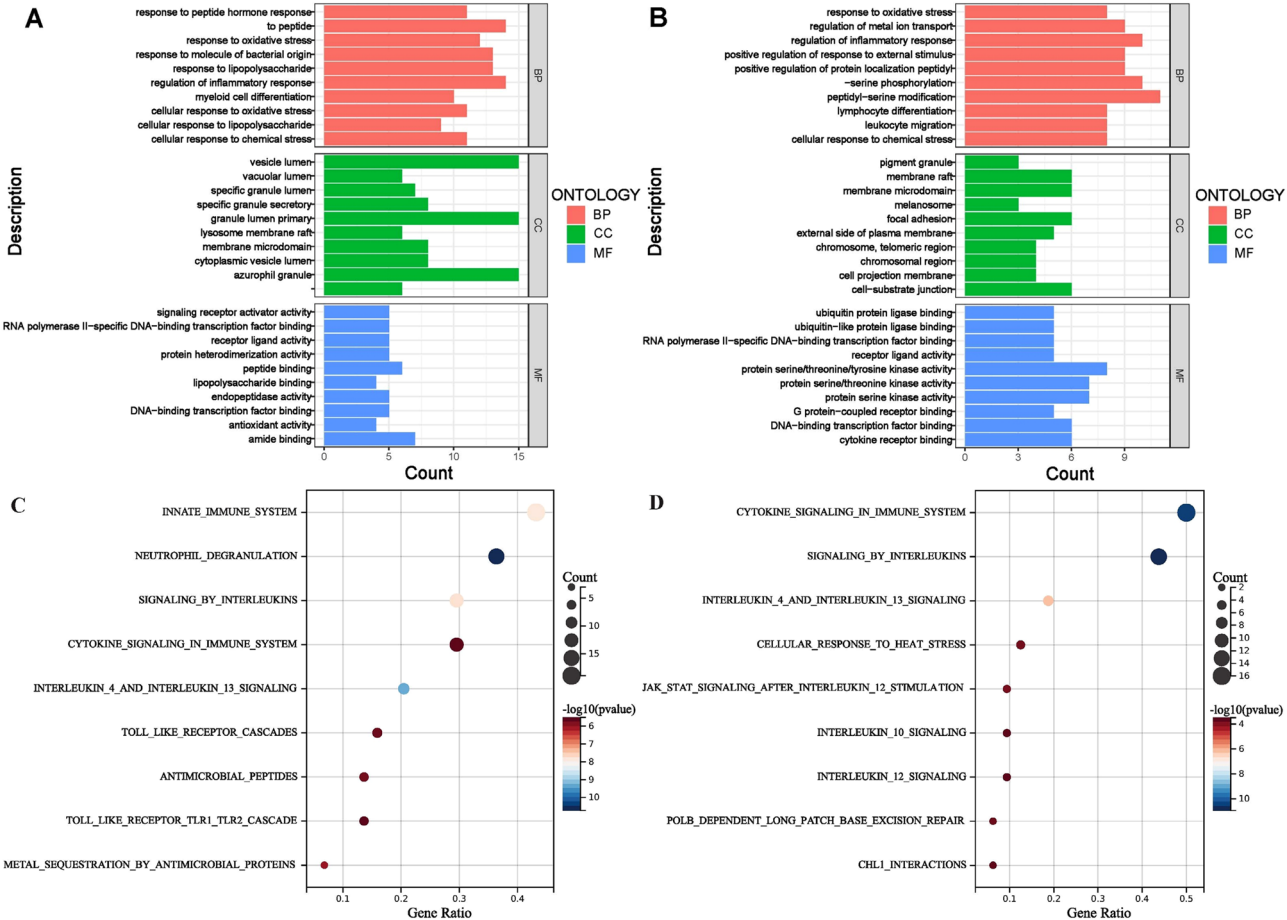
Functional enrichment analysis for differentially expressed oxidative stress genes (DEOSGS). (**A**) GO enrichment analysis for up-regulated DEOSGs (**A**) and down-regulated DEOSGs (**B**). Reactome enrichment analysis for up-regulated DEOSGs (**C**) and down-regulated DEOSGs (**D**).

GO enrichment analysis showed that up-regulated differentially expressed oxidative stress genes (DEOSGS) were significantly enriched in biological process related to regulation of inflammatory response, response to peptide, response to molecule of bacterial origin, response to lipopolysaccharide, response to oxidative stress (Fig. 3A) while down-regulated DEOSGS were significantly enriched in biological process related to peptidyl-serine modification, peptidyl-serine phosphorylation, regulation of inflammatory response, regulation of metal ion transport and response to oxidative stress (Fig. 3B). Reactome enrichment pathway analysis showed that up-regulated DEOSGS were significantly enriched in signaling pathways related to innate immune response, neutrophil degranulation, signaling by interleukins, cytokin signaling in immune system and interleukins 4/13 signaling (Fig. 3C) while down-regulated DEOSGS were significantly enriched in signaling pathways related to cytokin signaling in immune system, signaling by interleukins, interleukins 4/13 signaling, cellular response to heat stress and JAK STAT signaling after interleukins 12 stimulation (Fig. 3D).

### Gene set enrichment analysis for oxidative stress-related genes

**Figure 4:**
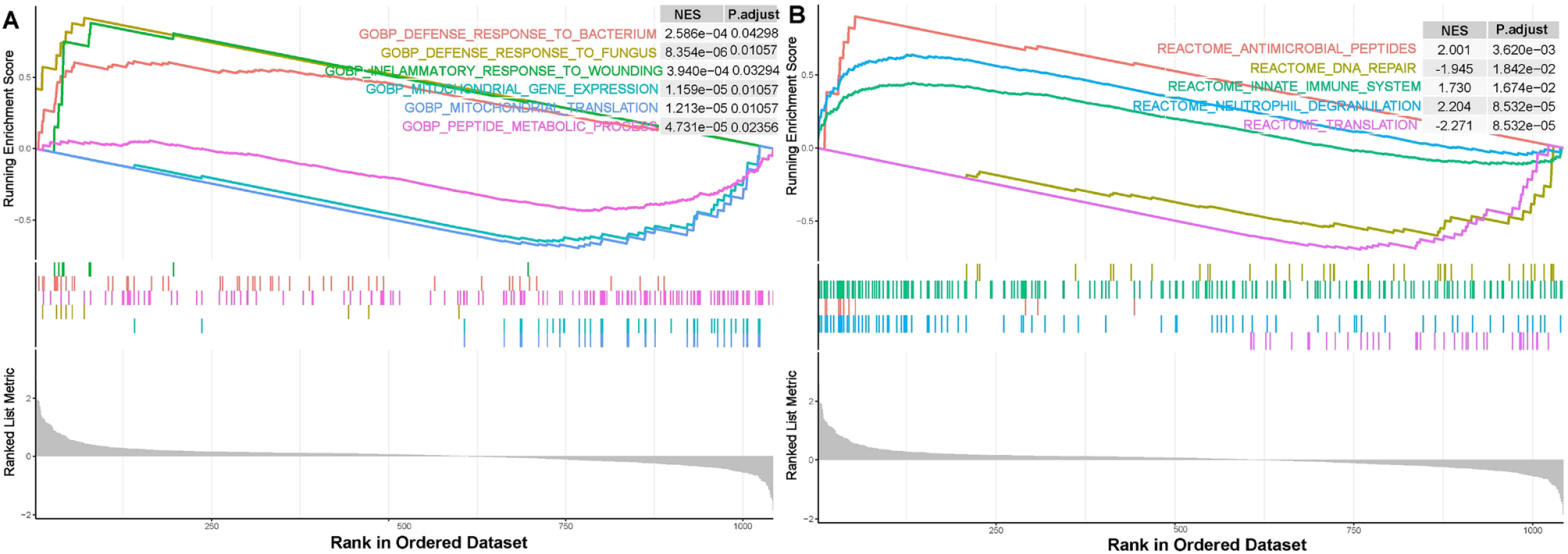
Gene set enrichment analysis (GSEA) using reference gene sets from GO database (**A**) and Reactome database (**B**) for oxidative stress-related genes (OSGs).

Expression information of oxidative stress-related genes (OSGs) in gene set GSE142255 was used for gene set enrichment analysis (GSEA). GSEA using c5.go.bp.v2023.1.Hs.symbols.gmt as gene sets showed that ACLF was significantly associated with biological process related to defense response to bacterium, defense response to fungus, inflammatory response to wounding, mitochondrial gene expression, mitochondrial translation and peptide metabolic process (Fig. 4A). GSEA using c2.cp.reactome.v2023.1.Hs.symbols.gmt as gene sets showed that ACLF was significantly associated with pathways related to innate immune system, neutrophil degranulation, antimicrobial peptide, DNA repair and translation (Fig. 4B).

### Immune infiltration analysis

**Figure 5:**
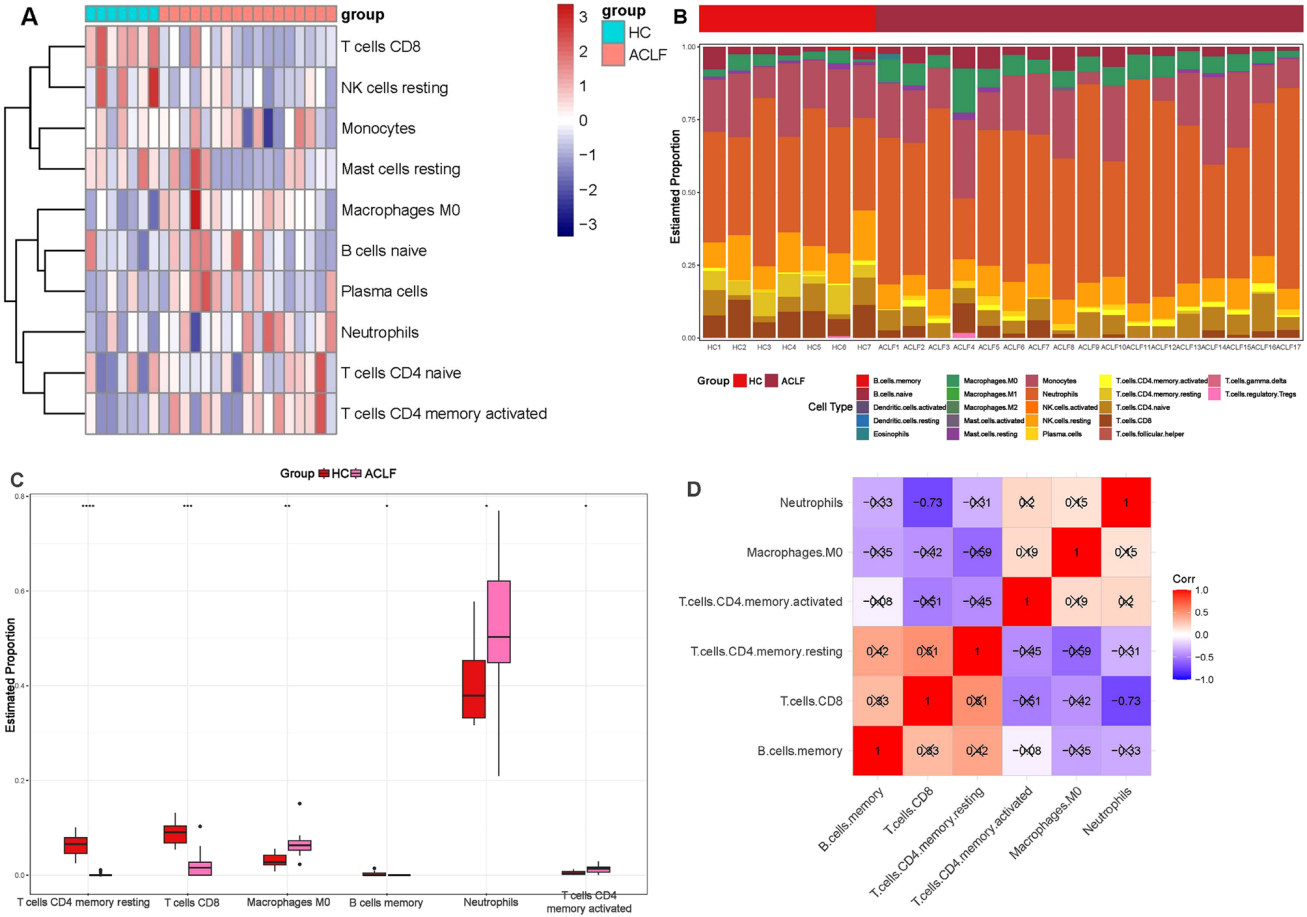
Immune infiltration analysis. Abundance (**A**) and proportions (**B**) of immune cells in samples from ACLF patients and healthy subjects. (**C**) Differentially infiltrating immune cells between ACLF patients and healthy subjects. (**D**) Correlations between differentially infiltrating immune cells. *HC* healthy control. Note: A cross indicates no statistical difference (P >0.05).

As shown in the functional enrichment analysis, DEOSGs and OSGs were strongly enriched in biological process and pathways indicated for immune response. Thus, we further investigated the relationship between gene expression in GSE142255 and immune cells infiltration using the CIBERSORT algorithm and visualized the result in heatmap (Fig. 5A,B). There is a significant difference in the abundance of six types of immune cells in samples from ACLF patients and healthy subjects (*P* < 0.05), including B cells memory, T cells CD4 memory resting, T cells CD4 memory activated, T cells CD8, macrophages M0 and neutrophils (Fig. 5C). Among the 6 differentially infiltrating immune cells, neutrophils were significantly and negatively correlated with CD8 T cells (*P* < 0.05) (Fig. 5D).

### Weighted gene co-expression network analysis (WGCNA)

**Figure 6:**
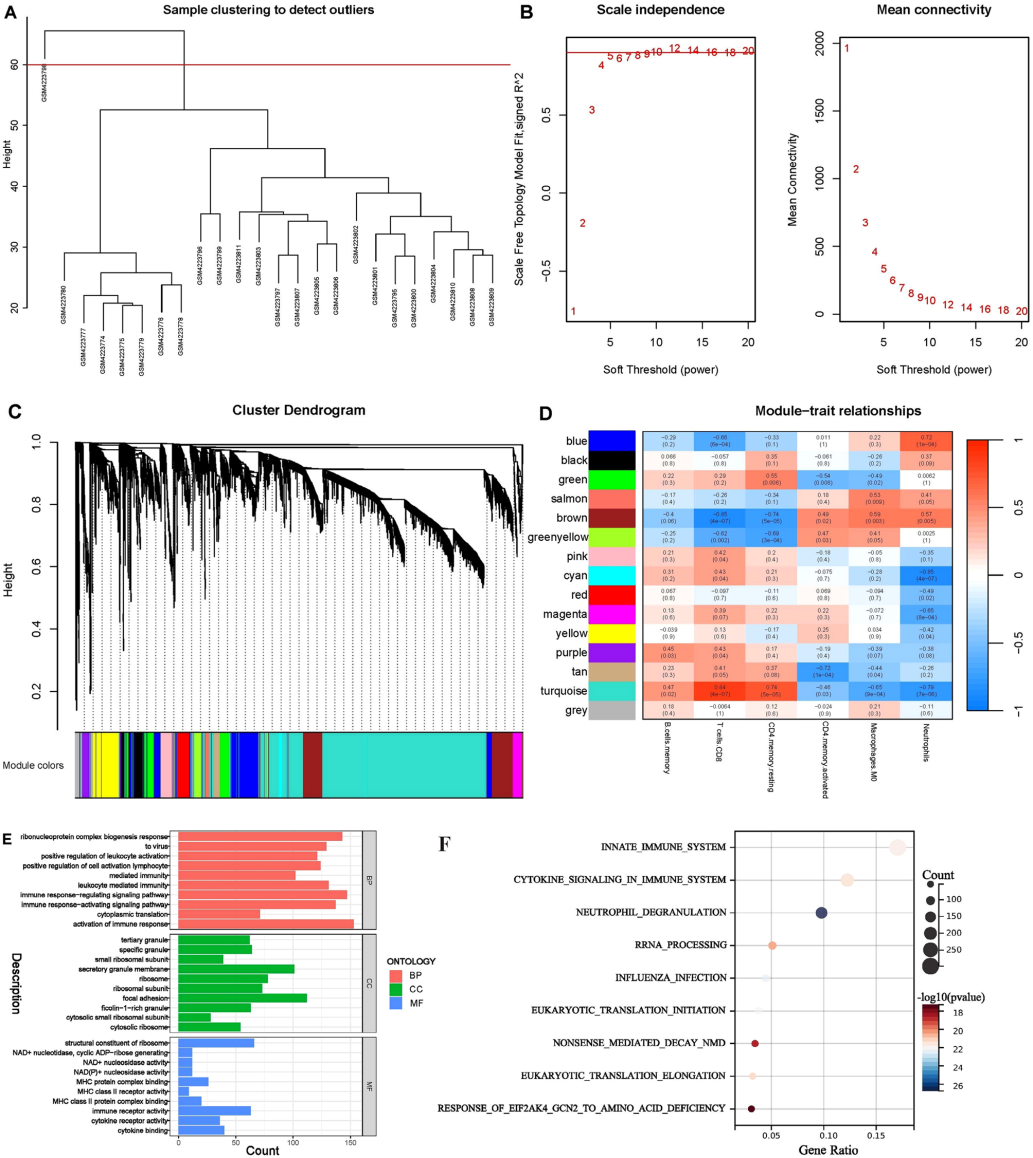
Weighted gene co-expression network analysis for gene set GSE142255 (**A–D**) and functional enrichment analysis for immune-related module genes (**E,F**). (**A**) Sample clustering to detect outliers. (**B**) Scale independence and mean connectivity. (**C**) Cluster dendrogram. (**D**) Module-trait relationships. (**E**) GO enrichment analysis for immune-related module genes. (**F**). Reactome enrichment analysis for immune-related module genes.

To identify the immune-related genes which were highly correlated with ACLF, we performed WGCNA. An outlier sample was identified and deleted (Fig. 6A). According to the scale independence and average connectivity, β = 10 was set as the soft threshold (Fig. 6B). On the basis of this power, 15 gene co-expression modules (GCMs) were generated (Fig. 6C). Among them, the brown, blue, cyan, tan and turquoise module demonstrated high correlation (correlation coefficient > 0.7, *P* < 0.001) with T cells CD8, CD4 memory resting cells and neutrophils (Fig. 6D). Therefore, these modules were regarded as key modules for subsequent analysis. A total of 3668 genes included in the key modules were subsequently named as immune-related modules genes. Functional enrichment analysis indicated that the modules genes were mainly enriched in pathways associated with immune response, activation or regulation (Fig. 6E,F).

### Construction of PPI network and screening of hub genes

**Figure 7:**
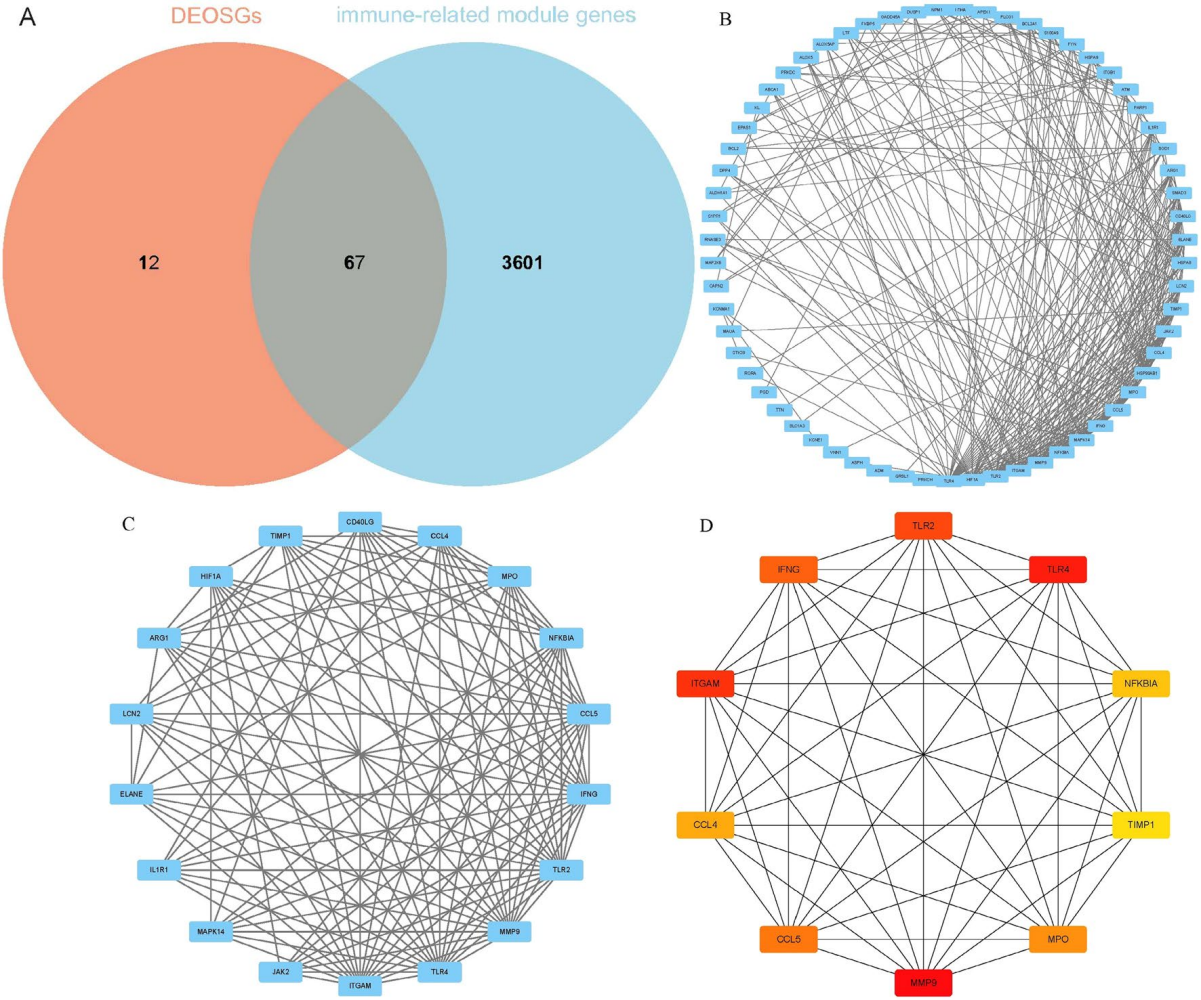
Protein–protein interaction (PPI) network and hub genes. (**A**) Immune-related DEOSGs. (**B**) PPI network constructed with immune-related DEOSGs (67 nodes and 294 edges). (**C**) Immune-related DEOSGs in cluster 1 of PPI network. (**D**) Hub genes screened out by MCC algorithm in cluster 1.

A total of 67 immune-related oxidative stress DEGS (OSDEGS) were identified by the intersection of 79 DEOSGs and 3668 immune-related module genes (Fig. 7A). After uploading the DEOSGs to STRING database, the protein–protein interaction (PPI) network was constructed and subsequently visualized with Cytoscape software (Fig. 7B). The most densely connected cluster (18 nodes and 117 edges) in PPI network, cluster 1, was identified by the MCODE plug-in in Cytoscape (Fig. 7C). The top 10 hub genes screened out by the MCC algorithm in cluster 1 were C–C motif chemokine 4 (CCL4), C–C motif chemokine 5 (CCL5), Integrin alpha-M (ITGAM), Interferon gamma (IFNG), Myeloperoxidase (MPO), Matrix metalloproteinase-9 (MMP9), NF-kappa-B inhibitor alpha (NFKBIA), Toll-like receptor 2 (TLR2), Toll-like receptor 4 (TLR4) and Tissue inhibitor metalloproteinase-1 (TIMP1) (Fig. 7D).

### Identification of key genes

**Figure 8:**
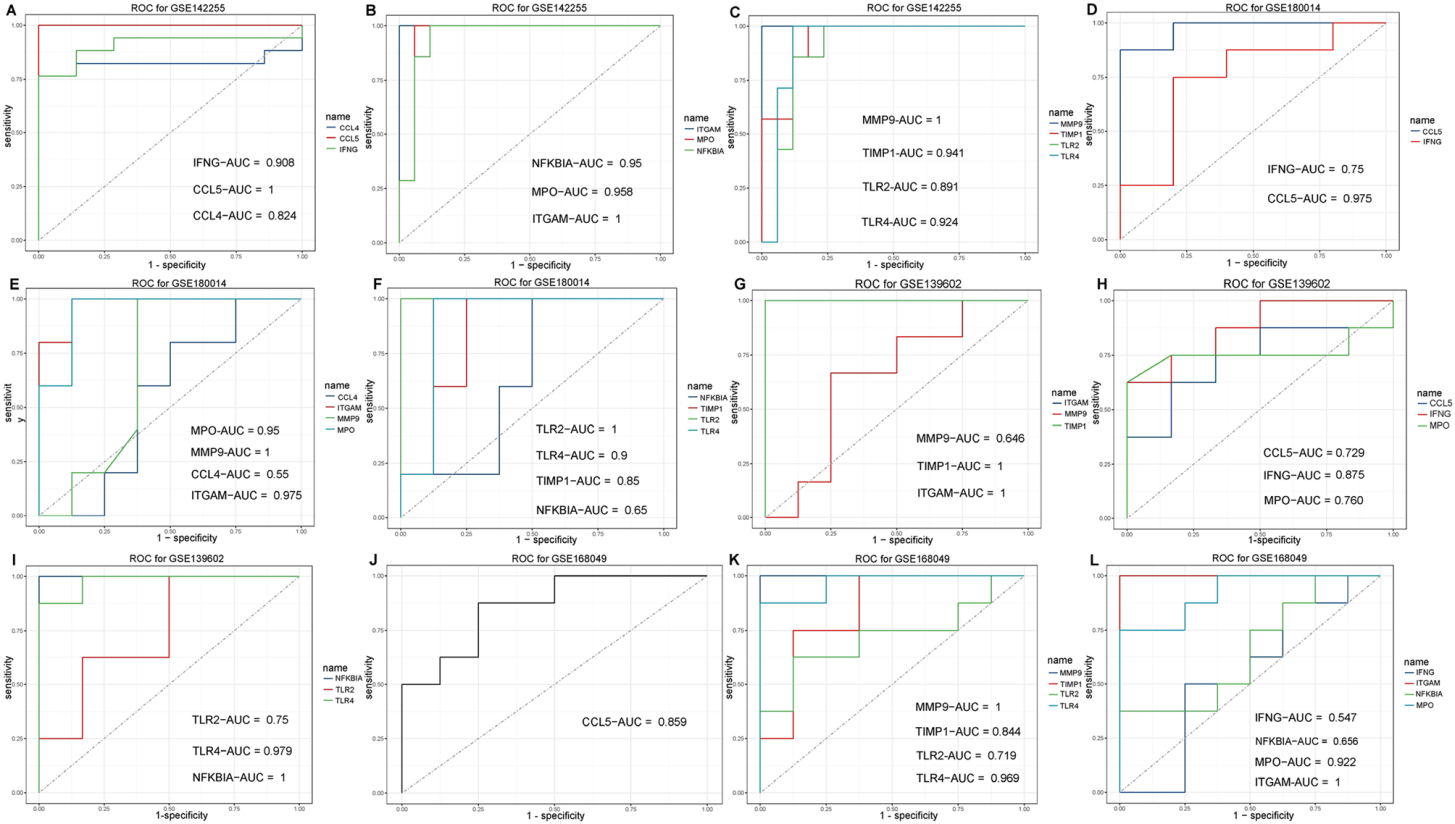
Receiver operating characteristic curve (ROC) analysis for hub genes in gene set GSE142255 (**A–C**), GSE180014 (**D–F**), GSE139602 (**G–I**) and GSE168049 (**J–L**).

To determine the diagnostic and prognostic value of hub genes for ACLF, we performed receiver operating characteristic curve (ROC) analysis. In data set GSE142255, the area under curve (AUC) values of all top 10 hub genes were > 0.7 (Fig. 8A–C). In data set GSE180014, hub genes with AUC > 0.7 include IFNG, MMP9, MPO, CCL5, ITGAM, TLR2, TLR4 and TIMP1 (Fig. 8D–F). In data set GSE139602, hub genes with AUC > 0.7 include IFNG, MPO, CCL5, ITGAM, NFKBIA, TLR2, TLR4 and TIMP1 (Fig. 8G–I). In data set GSE168049, hub genes with AUC > 0.7 include MPO, CCL5, MMP9, ITGAM, TLR2, TLR4 and TIMP1 (Fig. 8J–L). All together, a total of 6 hub genes (MPO, CCL5, ITGAM, TLR2, TLR4 and TIMP1) had consistently good diagnostic or prognostic value across four datasets and were thereafter named as key genes.

### Gene expression level of key genes

**Figure 9:**
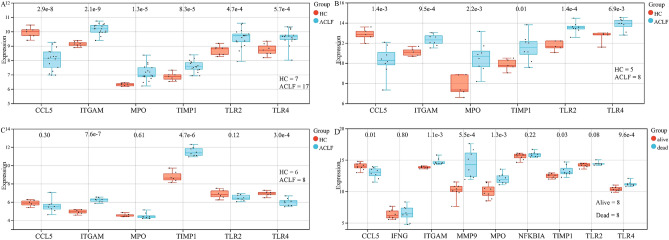
Gene expression level of key genes in gene set GSE142255 (**A**), GSE180014 (**B**), GSE139602 (**C**) and GSE168049 (**D**). HC,  healthy control.

To determine the difference in key genes expression between ACLF and HC, we performed independent samples t-test for these two groups. In data set GSE142255 and GSE180014, compared to the the gene expression level in HC group, CCL5 in the ACLF group was significantly down-regulated while the other 5 key genes were significantly up-regulated (*P* < 0.001) (Fig. 9A,B). In data set GSE139602 and GSE168049, 3 key genes (CCL5, MPO and TLR2) were found not significantly regulated (Fig. 9C,D).

### Functional enrichment analysis for key genes

**Figure 10:**
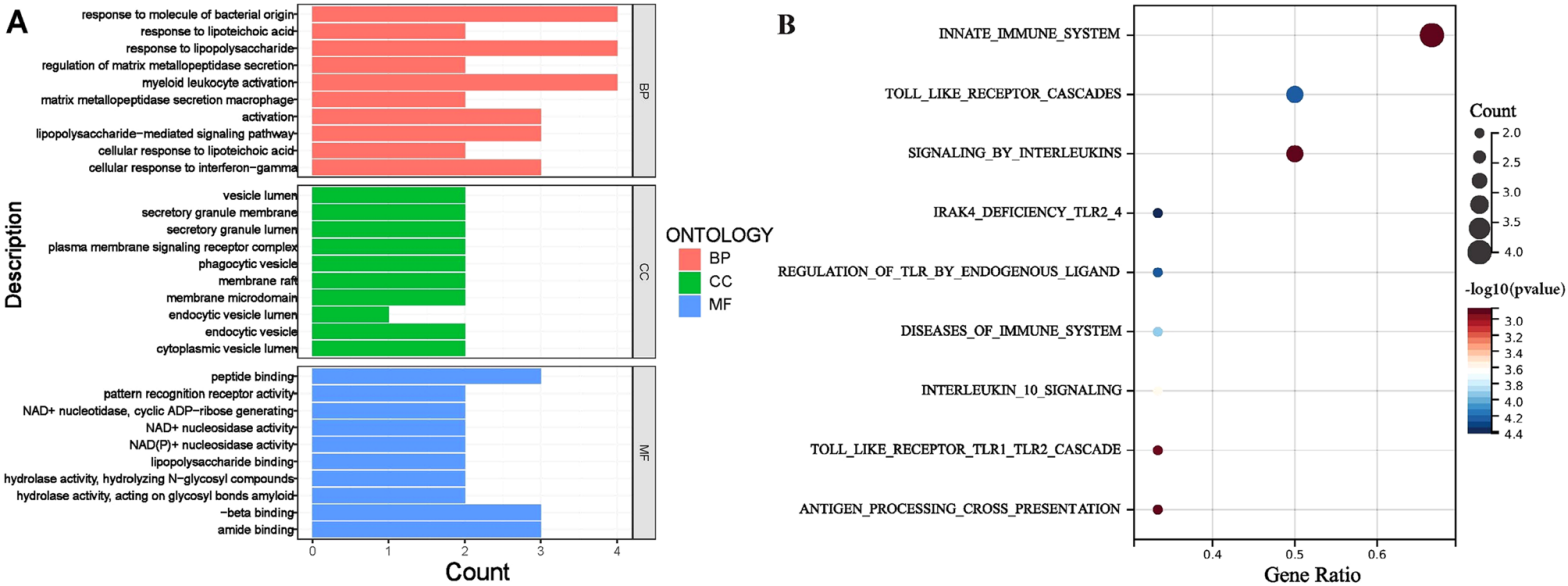
Functional enrichment analysis for key genes. (**A**) GO analysis. (**B**) Reactome analysis.

GO enrichment analysis showed that key genes were mainly enriched in biological process related to response to molecule of bacterial origin, response to lipopolysaccharide, myeloid leukocyte activation, macrophage activation, lipopolysaccharide-mediated signaling pathway, cellular response to interferon-gamma, regulation of matrix metallopeptidase secretion and cellular response to lipoteichioc acid (Fig. 10A). Reactome enrichment analysis showed that key genes were mainly enriched in pathways related to innate immune system, Toll like receptor cascades, signaling by interleukins, IRAK4 deficiency TLR2/4 and regulation of TLR by endogenous ligand, diseases of immune system, interleukin 10 signaling, Toll like receptor TLR1/TLR2 cascade and antigen processing cross presentation (Fig. 10B).

### Gene set enrichment analysis for key genes

**Figure 11:**
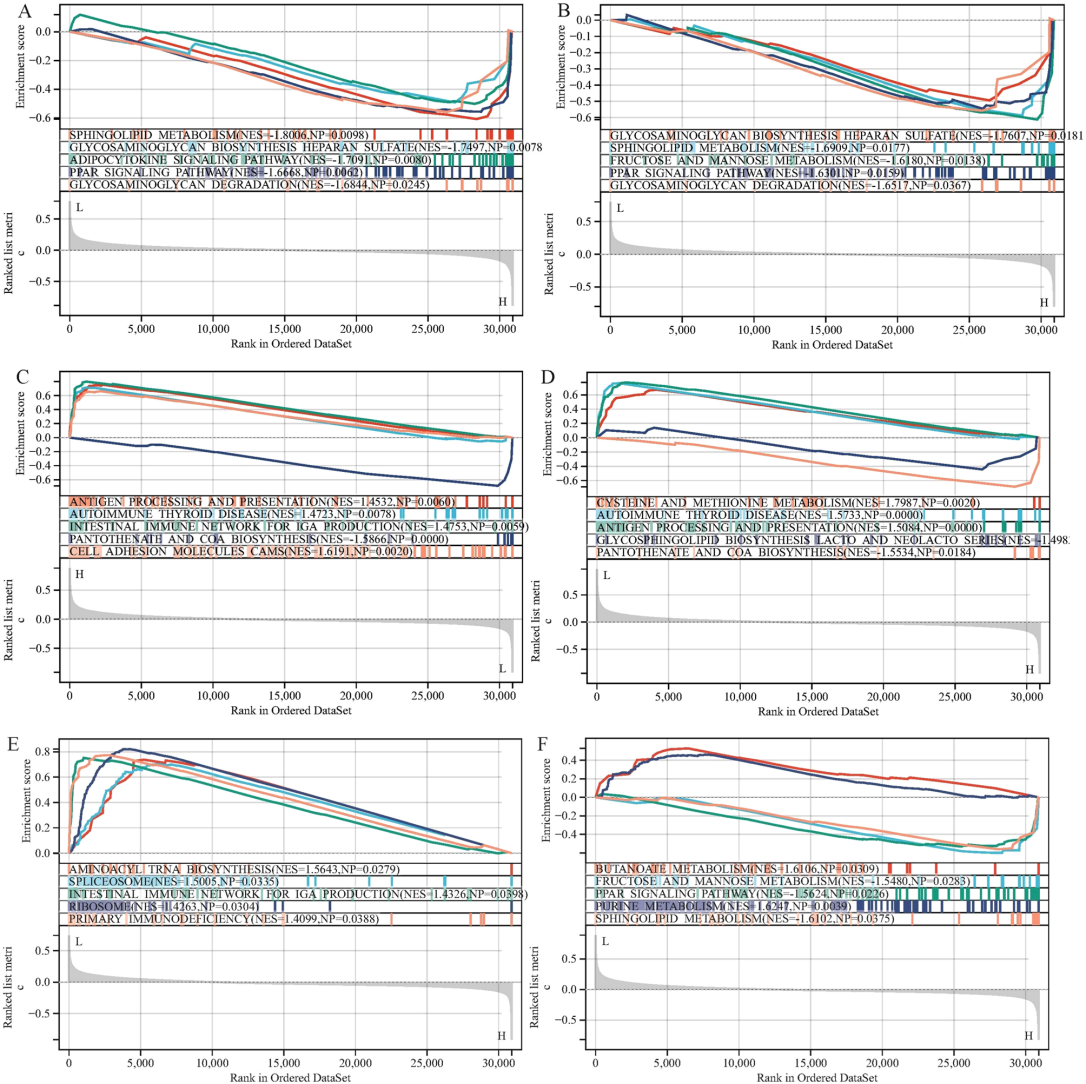
Gene set enrichment analysis (GSEA) for TLR2 (**A**), TLR4 (**B**), CCL5 (**C**), ITGAM (**D**), MPO (**E**) and TIMP1 (**F**).

To explore the potential function of key genes, we performed gene set enrichment analysis (GSEA) for single-gene with “c2.cp.kegg.v7.4.symbols.gmt” as the reference gene sets. The result showed that genes in cohorts with high expression of MPO, CCL5, ITGAM, TLR2, TLR4 and TIMP1 were highly enriched in immune and metabolism-related pathways, such as antigen processing and presentation, autoimmune thyroid disease, intestinal immune network for IGA production, primary immunodeficiency, sphingolipid metabolism, fructose and mannose metabolism, cysteine and methionine metabolism, butyrate metabolism, purine metabolism, glycosaminoglycan biosynthesis heparan sulfate, PPAR signaling pathway, glycosaminoglycan degradation, glycolipid biosynthesis lactose and new lactose series, pantothenate and coenzyme a biosynthesis and aminoacyl ribonucleic acid biosynthesis (Fig. 11A–F), indicating that the key genes might participate in the process of immune response and biological metabolism.

### Correlation between key genes and their association with immune cells

**Figure 12:**
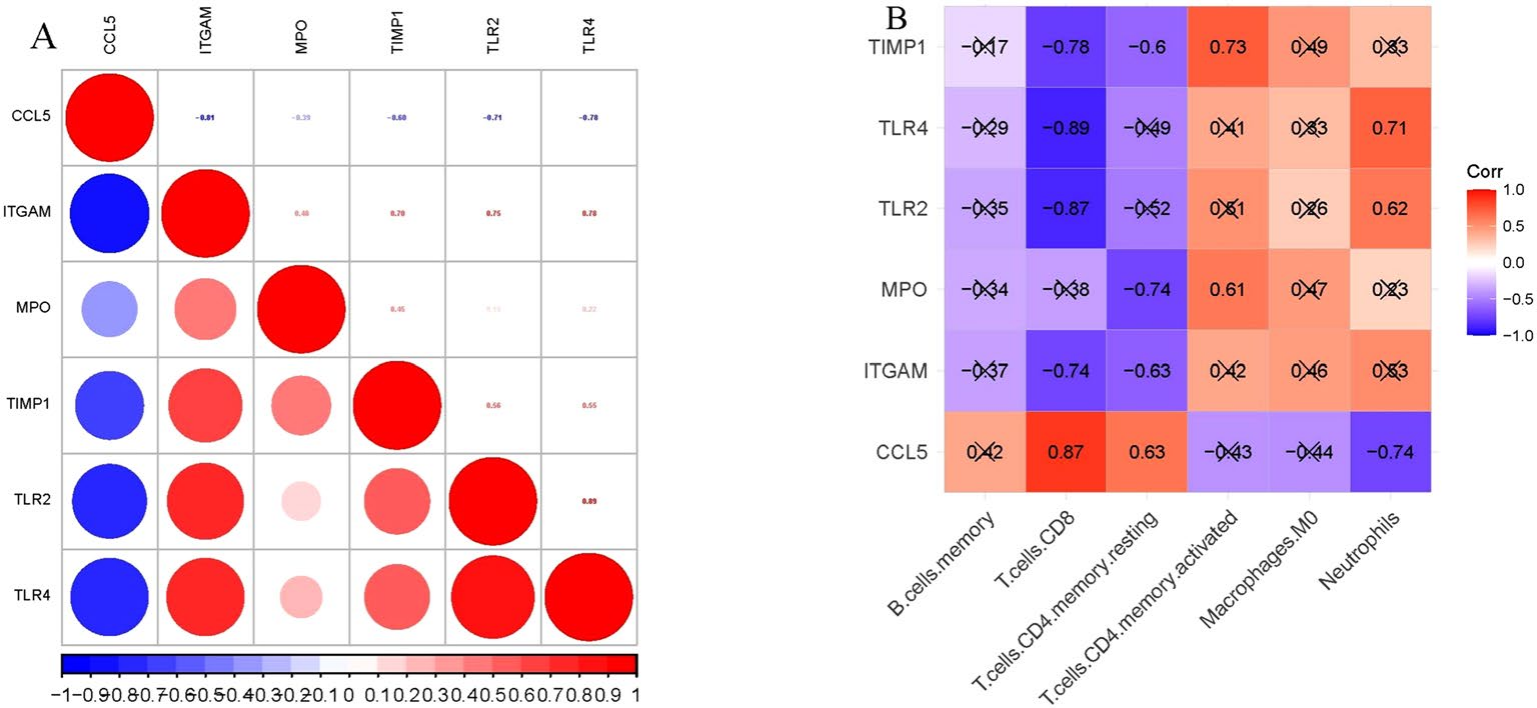
Correlation between key genes (**A**). Correlation between key genes and differentially infiltrating immune cells (**B**). Note: A cross indicates no statistical difference (P >0.05).

To determine the correlation between key genes and their association with immune cells infiltration, we performed spearman correlation analysis. The result showed that CCL5 was negatively correlated with the other 5 key genes which were positively correlated with each other (Fig. 12A); all key genes except CCL5 (positive) and MPO (no significance) had significantly negative correlation with CD8 T cells; MPO and TIMP1 had significantly negative and positive correlation with resting memory CD4 T cells and activated memory CD4 T cells, respectively. CCL5 and TLR4 had negative and positive correlation with neutrophils, respectively (Fig. 12B).

### Identification of transcription factors and miRNAs regulating key genes

**Figure 13:**
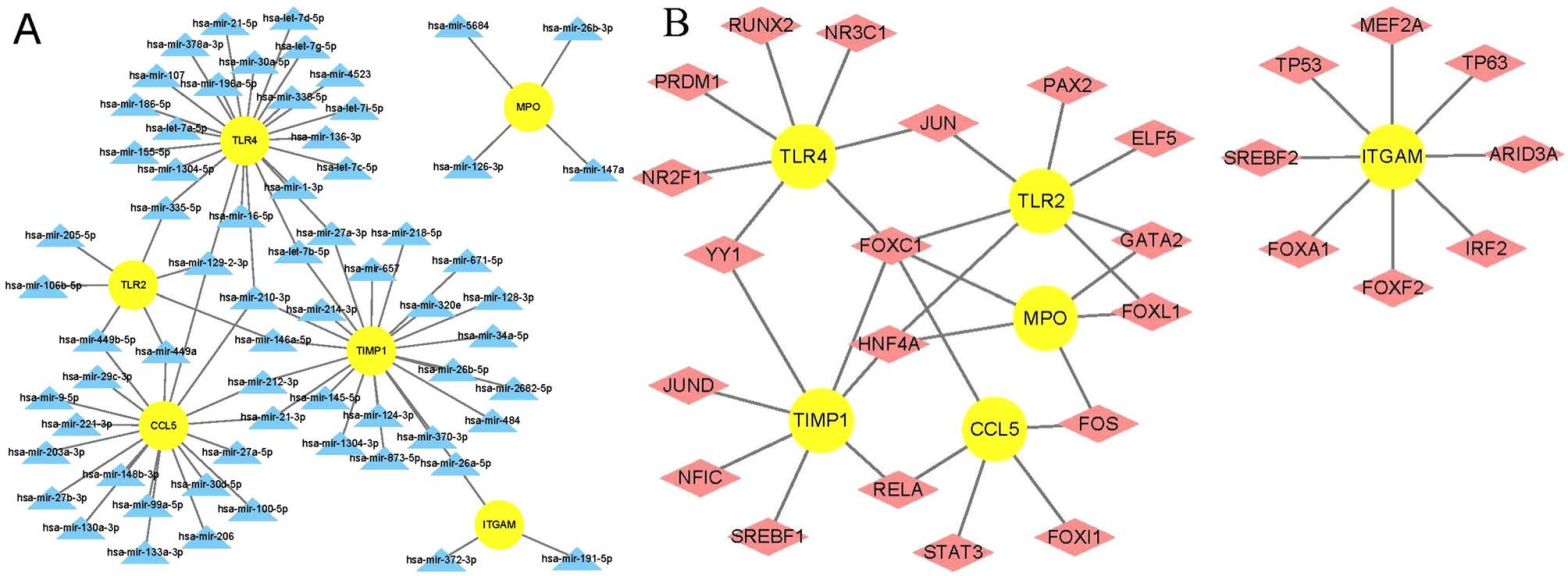
Regulatory networks for key genes. (**A**) Micro RNA–key gene interaction network. (**B**) Transcription factor–key gene interaction network. Note: The sky blue triangle indicates micro RNA, yellow circle indicates key genes, and wine red diamond indicates transcription factors.

To determine the regulatory impact of miRNAs and TFs on expression of key genes at the transcriptional level, we obtained the interaction networks of miRNAs and TFs with key genes and visualized them with Cytoscape software. A total of 23, 22, 19, 7, 4 and 3 miRNAs interacted with TLR4, TIMP1, CCL5, TLR2, MPO and ITGAM, respectively (Fig. 13A). A total of 8, 7 and 5 TFs interacted with ITGAM, TLR2/TLR4/TIMP1 and MPO/CCL5, respectively (Fig. 13B). Both miRNAs (hsa-mir-129-2-3p and hsa-mir-210-3p) interacted with 3 key genes and both TFs (FOXC1 and HNF4A) interacted with 4 key genes, indicating that these miRNAs and TFs might have a closer interaction with the key genes.

### Identification of potentially effective drugs

**Figure 14:**
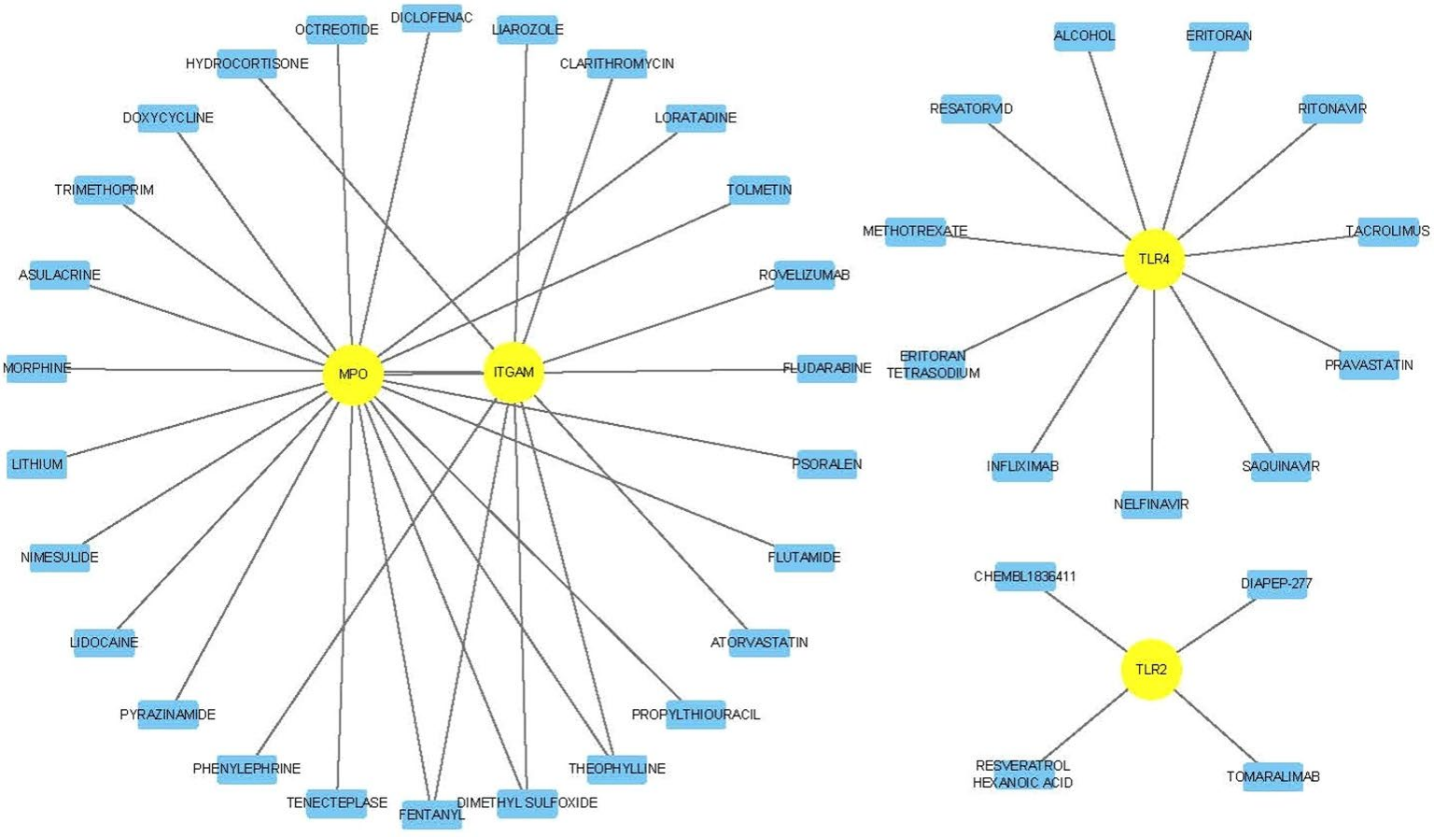
Drug–key genes interaction network. Note: The sky blue rectangle indicates drugs, and yellow circle indicates key genes.

To explore potentially effective drugs targeting the key genes, we obtained the gene-chemical interaction network from drug–gene interactions database (DGIdb) and visualized them with Cytoscape software (Fig. 14). Among the 6 key genes, TLR2, ITGAM, TLR4 and MPO had intersections with 4, 10, 11 and 19 drugs/chemicals, respectively. Among the 41 drugs, fentanyl, dimethyl sulfoxide and theophylline had intersections with two key genes (MPO and ITGAM).

## Discussion

Acute-on-chronic liver failure (ACLF) is associated with a poor prognosis, limited therapeutic options and incompletely understood pathogenesis. There is an urgent need to further reveal the pathogenesis underlying the occurrence and progression of ACLF in order to develop effective targeted drugs and accurately predict its prognosis. In this study, we identified six immune and oxidative stress-related genes proven to be associated with the diagnosis and prognosis of ACLF with the integrated bioinformatics method, which might provide new perspectives for the molecular targeted therapy and prediction of prognosis in ACLF.

Development of ACLF is highly associated with inflammation and immune dysfunction. Functional enrichment analysis for differentially expressed genes (DEGs) between patients with ACLF and healthy controls indicated that the ACLF was highly associated with an activated inflammatory response, enhanced innate immune response and impaired adaptive immune response. For instance, signaling pathways indicating inflammatory and innate immune responses, including Toll-like receptor (TLR) and inflammatory signaling^19^, monocytes and monocyte surface signature were up-regulated in ACLF while pathways indicating adaptive immune response, including T cell surface signature, activation, signaling and differentiation, NK cells and MHC-TLR7-TLR8 cluster were down-regulated in ACLF. Furthermore, as indicated in the immune cells infiltration analysis, activated memory CD4 T cells^20^, M0 macrophages and neutrophils involved in the acute inflammatory response were significantly up-regulated in ACLF, while memory B cells^21^, memory resting CD4 T cells^20^ and CD8 T cells^22^ involved in the adaptive immune response were significantly down-regulated in ACLF. Taken together, our study supported the view that the development of ACLF is highly associated with an intensive systemic inflammation and immune dysfunction characterized by coexistence of enhanced innate immune response and paralyzed adaptive immune response^2,3^.

Oxidative stress was highly involved in the development of ACLF. As indicated in the gene set enrichment analysis (GSEA) for oxidative stress related genes (OSGs), ACLF was positively associated with oxidative stress-related metabolic process, such as collagen degradation, degradation of extracellular matrix, activation of matrix metalloproteinases, NFE2L2 regulated antioxidant detoxification enzyme and metabolic disorder of biological oxidation enzyme. In addition, ACLF was also positively associated with inflammatory response and innate immune response, such as inflammatory response to wounding, acute inflammatory response, antimicrobial peptide, innate immune system, neutrophil mediated immunity, defense response for bacterium, cell surface toll like receptor signaling pathway, regulation of leukocyte mediated cytotoxicity. By contrast, ACLF was negatively associated with adaptive immune response, such as regulation of lymphocyte mediated immunity. Therefore, our study indicated that oxidative stress, along with inflammation and immune dysfunction, might jointly participate in the development of ACLF.

### We identified 6 oxidative stress-related key genes associated with the diagnosis and prognosis of ACLF

Based on the PPI network and subsequent internal and external validation, 6 hub genes with consistent good diagnostic and prognostic values across the four datasets were named as key genes, including CCL5, ITGAM, MPO, TLR2, TLR4 and TIMP1. As an inflammatory chemokine stored in post-naive CD8+ T cells, CCL5 is exocytosed rapidly after TCR ligation to selectively recruit the receptor-bearing leukocytes to sites of injury or pathogens^23^. Oxidative stress can modulate the expression of CCL5 in an oxidant-dependent signaling pathway^24^. ITGAM is mainly expressed on phagocytic cells and natural killer (NK) cells as a membrane receptor mediating the phagocytosis for certain bacteria after ligation with its carbohydrates or lipopolysaccharides (LPS)^25,26^. MPO is associated with both inflammatory response and oxidative stress due to its location in neutrophils and its role in catalyzing the formation of oxidizing agents under the stimulation of pro-inflammatory mediators^27^. TLR2 and TLR4 participate in the process of innate immune response by recognizing the bacterial lipoproteins and LPS, respectively^19^. As a sensor of oxidation-associated molecular patterns^28^, TLR2 may act as a bridge between oxidative damage and various pathological injury, such as angiogenesis^28^ and excessive complement activation in retinal degenerative disease^29^. TLR4 primes the responsiveness of innate immune cells when recruited to the plasma membrane in macrophages under oxidative stress^30^ and plays a critical role in the LPS-induced oxidative stress and mitochondrial disorder^31^. TIMP1 has been shown favoring extracellular matrix (ECM) deposition and fibrotic scarification in process of liver fibrosis by binding to the catalytic zinc cofactors of several matrix metalloproteinases (MMP2, MMP9 and MMP13) attenuating their constitutive matrix degrading potential^32^. Oxidative stress and reactive oxygen species (ROS) may be important factors leading to liver fibrosis^33^. Therefore, there might be a correlation between TIMP1 and oxidative stress, requiring further research. Functional enrichment analysis indicated that key genes were highly associated with inflammation, immune response and metabolic process. Immune cell infiltration analysis indicated that all the keys genes were positively or negatively correlated to the differentially infiltrating immune cells. In summary, key genes well reflected the biological functions of immune related DEOSGs, which were highly associated with the occurrence and progression of ACLF. Therefore, the good diagnostic and prognostic value of key genes for ACLF might be explained.

In addition, we constructed a gene regulatory network and explored some potentially effective molecular targeted drugs for ACLF. Transcription factors (TFs) are a type of protein complex that plays an important role in the regulation of gene transcription through recognizing specific DNA sequences^34^. MicroRNAs (miRNAs) are small non-coding RNA molecules involved in post-transcriptional gene regulation by binding to one or more sites of mRNA transcription sequences and inhibiting their translation or by regulating the TFs, with subsequent effects on various cellular and biological processes under normal and pathological conditions^35^. In the miRNA-gene and TF-gene interaction network, two miRNAs (hsa-mir-129-2-3p and hsa-mir-210-3p) and two TFs (FOXC1 and HNF4A) interacted with most key genes, indicating that they might be involved in the regulation of key genes. In addition, we identified 41 drugs potentially targeting the key genes, among which fentanyl, dimethyl sulfoxide and theophylline all targeted two key genes, which means that they might become the candidates for molecular targeted therapy.

The present study has some limitations that should be mentioned. First, the sample size is relatively small. Nevertheless, we validated the diagnostic and prognostic value of key genes in four independent datasets. Therefore, our results might have a certain degree of credibility. Second, all results obtained in this study were only based on the microarray or RNA-seq data from GEO database. Further in vitro and in vivo experimental verification is still required. Third, the retrospective design of this study would inevitably induce bias, which might affect the reliability of results. Confirmation for our findings from strictly designed prospective studies is still required.

In conclusion, the present study explored the role of oxidative stress in the occurrence and progression of ACLF using the integrated bioinformatics methods. We identified six immune and oxidative stress related genes which were highly associated with the diagnosis and prognosis of ACLF. In addition, we constructed a regulatory network for key genes and explored some drugs targeting the key genes. This study deepened our understanding of the impact of oxidative stress on the pathogenesis and prognosis of ACLF, and provided more insights into the effective molecular targeted therapy strategies. More prospective studies and further experiments are still required to validate our results.

## Data Availability

Datasets (GSE142255, GSE180014, GSE139602 and GSE168049) used in this study are available in Gene Expression Omnibus (GEO) repository (http://www.ncbi.nlm.nih.gov/geo/).
